# Evidence-based indicators for the measurement of quality of primary care using health insurance claims data in Switzerland: update of the SQUIPRICA working group

**DOI:** 10.1186/s12913-022-07893-8

**Published:** 2022-05-11

**Authors:** Eva Blozik, Renato Farcher, Sereina M. Graber, Carola A. Huber, Swiss Quality Indicator for Primary Care (SQIPRICA) Working Group, Jakob Burgstaller, Corinne Chmiel, Felix Huber, Philippe Luchsinger, Leander Muheim, Oliver Reich, Thomas Rosemann, Martin Scherer, Felix Schnweuwly, Oliver Senn, Daniel Tapernoux

**Affiliations:** 1 Department of Health Sciences, Helsana Group, P.O. Box, Zürich, Switzerland; 2 Institute of Primary Care, University of Zürich and University Hospital of Zürich, CH-8091 Zürich, Switzerland

**Keywords:** Quality indicator, Quality assessment, Quality measurement, Claims data, Health insurance, Evidence-based, Consensus process

## Abstract

**Background:**

The level of quality of care of ambulatory services in Switzerland is almost completely unknown. Since health insurance claims are the only nationwide applicable and available data source for this purpose, a set of 24 quality indicators (QI) for the measurement of quality of primary care has been previously developed and implemented. The present paper reports on an evidence-based update and extension of the initial QI set.

**Methods:**

Established pragmatic 6-step process based on informal consensus and potential QI extracted from international medical practice guidelines and pre-existing QI for primary care. Experts rated potential QI based on strength of evidence, relevance for Swiss public health, and controllability in the Swiss primary care context. Feasibility of a preliminary set of potential new QI was tested using claims data of persons with basic mandatory health insurance at one of the largest Swiss health insurers. This test built the basis for expert consensus on the final set of new QI. Additionally, two diabetes indicators included in the previous QI set were re-evaluated.

**Results:**

Of 23 potential new indicators, 19 of them were selected for feasibility testing. The expert group consented a final set of 9 additional QI covering the domains general aspects/efficiency (2 QI), diagnostic measures (1 QI), geriatric care (2 QI), osteoarthritis (1 QI), and drug safety (3 QI). Two pre-existing diabetes indicators were updated.

**Conclusions:**

Additional QI relating to overuse and intersectoral care aspects extend the options of measuring quality of primary care in Switzerland based on claims data and complement the initial QI set.

## Background

Quality improvement is not possible without quantitative quality assessment [1] Despite a variety of initiatives that aim to increase quality of primary care in Switzerland [2–5]. including certification measures, in-house medical guideline development, or quality circles, measures to raise transparency on the level of quality of care of ambulatory services in Switzerland were lacking. Therefore, in 2018, a first set of nationwide applicable quality indicators (QI) has been presented [6]. These QI are calculated based on information from health insurance claims data, the single data source that is nationwide pre-existing and available in a standardised and equivalent format. The intention of the project was to propose a set of rigorously developed and publicly available QI based on evidence from national and international guidelines and pre-existing QI including assessment of local public health relevance and patients´ and consumers´ perspectives. The central idea was to continuously evaluate, refine and expand the proposed QI.

The aim of the present paper is to describe the update process and to present additional QI consented by the expert group. Results of this informal update and expansion process are of immediate relevance for the local health system: 7 indicators of the initial indicator set are currently in different implementation phases in the context of contracts between health insurances and health service providers and are therefore directly impacting health services provision and healthcare provider reimbursement schemes in Swiss routine primary care [7]. In general, on the national regulatory level relating to basic mandatory health insurance in Switzerland, most recently a new legislation entered into force which requires healthcare providers and insurers to conclude national agreements on quality development so that quality indicators for the ambulatory sector are or increasing importance for various stakeholders in the Swiss healthcare system [6].

## Methods

### Context of the study

Health insurance is obligatory for all persons living in Switzerland. The basic health insurance catalogue is similar across all patient groups and regions and includes all outpatient or inpatient medical services deemed appropriate, medically effective, and cost-effective. Supplementary hospital insurance in Switzerland is available if individuals wish further comfort such as semiprivate or private ward. There are currently about sixty insurance companies offering basic health coverage in Switzerland, and they provide various premiums and health plans from which Swiss residents are free to select [8]. Registering with a GP is generally not required, and residents insured in the standard insurance plan have free choice among GPs. However, persons are free to choose managed care plans (e.g. integrated care plans, telephone triage plans, capitated and non-capitated plans) in which they need to contact a specific primary care provider before seeking care with other healthcare providers. Helsana is one of the largest Swiss health insurances covering about 15% of the Swiss population from all parts of the country.

### Study protocol

The established multi-stage development process has been established previously [6]. Identification of potential new QI was based on a literature review for guidelines and pre-existing QI specific for primary care/ for the primary care setting. The following sources were used for the search:

- guidelines of the German association of primary care and family medicine (Deutsche Gesellschaft für Allgemeinmedizin und Familienmedizin, DEGAM): all guidelines updated and published after 2018 (guidelines published before 2018 were included in development of the initial QI set) [9]
- German National Disease Management Guidelines (Nationale VersorgungsLeitlinien, NVL): all guidelines updated after 2018 (guidelines published before 2018 were included in development of the initial QI set) [10]
- QISA (QI for primary care, developed by the AQUA Institute) indicators: all QI updated after 2018 (QI published before 2018 were included in development of the initial QI set) [11]
- Choosing Wisely recommendations from national medical specialty societies in the U.S.A. [12]
- Smarter Medicine Initiative: top 5 recommendations from medical specialty societies in Switzerland [13].
- The NICE menu of indicators for primary care [14].
- ACOVE-3 (Assessing Care of Vulnerable Elders) of the RAND Initiative [15]
- European Commission Report on Tools and Methodologies to Assess Integrated Care in Europe [16]

Guidelines / QI for use in other settings such as ambulatory hospital or inpatient setting were excluded. In a first step, we extracted all recommendations for or against specific medical interventions. Secondly, this list of potentially eligible items for QI was checked for operationalisability on Swiss health insurance claims data. For example, information on indication or anamnestic data collection is lacking in Swiss health insurance claims data and such indicators had to be excluded. In a third step, the previously established SQIPRICA (Swiss Quality Indicator for Primary Care group) including independent multidisciplinary experts from primary care, public health, and health economics and patient and consumer representatives rated the list of potential QI. Criteria for rating were relevance for public health, clarity of definition, influence on measured aspect of care, risk of undesired effects, and strength of evidence [6].

Experts were asked to rate the potential indicators according to a 4 point Likert scale (1 = incorrect; 2 = rather incorrect; 3 = rather correct; 4 = fully correct). For the aspect risk of undesired effects, they were asked to answer yes or no.

As a fourth step, there was an online workshop to discuss rating results and to reach consensus on a preliminary set of QI qualifying for a first practical test.

As a fifth step, based on claims data of 924′839 adult persons with basic mandatory health insurance in the year 2019 potential QI were provisionally calculated on a pilot basis. The data base included information on demographics and reimbursed health care utilization, including number of consultations and information on drugs, laboratory and imaging tests and type of the treated health care provider. Specifically, we tested whether it was possible to apply potential QI using claims data. The proportion of persons presenting with the QI at interest was calculated, stratified and operationalized by QI specific criteria defined by the expert group such as age and gender stratification. Continuity of care was operationalized using the Usual Provider Continuity Index (UPC) [17]. Analyses were performed using the statistical software R, version 4.0.2 (R Foundation for Statistical Computing, Vienna, Austria). As a final (sixth) step a second online workshop with the expert group was performed to discuss the results of the feasibility test and to reach consensus about the final set of additional QI.

Based on stakeholder feedback to the SQIPRICA group, for two of the four QI relating to diabetes (QI #19 and # 20 based on [6]), an update process was initiated: First, international sources as listed above and additional international guidelines were systematically searched for explicit recommendations for or against testing of lipid profile and renal status in specific diabetes patient populations. Then results were discussed at both expert group workshops. Consensus on update of the corresponding QI was built at the second workshop.

### Ethical approval

The exploratory statistical analyses of the feasibility test complied with the Swiss Federal Law on data protection. All data were anonymized and de-identified prior to the performed analysis to protect the privacy of patients, physicians, and hospitals. According to the national ethical and legal regulation, an ethical approval was not needed because the data were retrospective, pre-existing, and de-identified. Since data was anonymized, no consent of patients was required.

## Results

We extracted guideline recommendations and QI from 3 National Disease Management Guidelines, 4 QiSA indicator sets, 21 DEGAM primary care S1, S2 and S3 guidelines, 231 Choosing Wisely recommendations, 14 Smarter Medicine recommendations, 17 QI sets from the EU Commission report, 24 ACOVE and 42 NICE indicator sets. We excluded duplicates, services that are not part of the basic mandatory health insurance package in Switzerland and measures that cannot be mapped using claims data such as details of clinical processes, decision making, or communication that are not relevant for reimbursement. A list of 23 potential new QI was sent to the expert group for rating of relevance for public health, clarity of definition, influence on measured aspect of care, risk of undesired effects, and strength of evidence. Overall, there were few discrepancies related to the rating across the group. All potential QI were assigned high values for the aspect “relevance for public health” (mean and median 3 = “rather correct”).

Twenty-one indicators were rated “rather correct” or “fully correct” for all or the majority of the rating criteria. Two indicators failed rating: “dispersion between the health care providers” and “GP emergency visit” were considered to lack influenceability by primary health care providers, clarity of definition, and strength of evidence and were thus excluded by the expert group. Based on in-depth discussions during the workshop, several additional indicators were excluded from the list of potential QI because the expert group questioned that the indicator can be validly constructed based on information available in claims data (see Table 1). The first online workshop resulted in a set of 19 preliminary new indicators qualifying for the feasibility test covering the domains general aspects/ efficiency (4 candidate QI), laboratory testing (4), screening (1), imaging (2), geriatric care (1), osteoarthritis (1), and drug safety (5).

**Table 1:** Candidate quality indicators excluded in the first and/ or second expert workshop

Category	Potential indicator	Potential operationalization	Reason for exclusion
**Indicator excluded in the first expert workshop**			
General aspects/ efficiency	Avoidable specialist visits	Proportion of personswith diabetes or hypertension unnecessarily transferred to a specialist for an uncomplicated cause (only considered diagnos: diabetes and hypertension). Estonia national guideline. Uncomplicated is based on an expert evaluation of all diagnosis codes.	The operationalization of the QI is based on ambulatory diagnoses (not available in Swiss health insurance claims data. Approximation using Pharmacy Cost Groups seems inappropriate.
General aspects/ efficiency	Dispersion between providers	1) Ratio of primary care professionals (e.g. GPs) to specialists 2) Modified, modified continuity index (MMCI): This index focuses on the dispersion between providers and is based on the number of caretakers and number of visits only.	Redundancy: similar to the existing indicator (“Number of different primary care physicians consulted by an individual insured person”).
General aspects/ efficiency	Emergency GP visits	1) Rate of emergency visits for adults 2) Number of emergency visits	Based on health insurance claims no differentiation between emergency and non-emergency possible.
**Indicator excluded in the second expert workshop**			
General aspects/ efficiency	Medication after hospital discharge	1) Proportion of persons with polymedication after vs. before hospitalization 2) Proportion of persons with PIM prescription after vs. before hospitalization	Limited influence of the GP on hospital medication prescription. Measures quality of hospital care/hospital processes.
Laboratory testing	Vitamin B12 testing	Proportion of persons who received one or more vitamin B12 test	Information about the indication for vitamin B12 testing/ symptoms is missing in the claims data.
Laboratory testing	Ferritin testing	Proportion of persons who received 1 or more ferritin test	Information about the indication for ferritin testing / symptoms is missing in the claims data.
Laboratory testing	Complex lymphocyte panel	Proportion of persons who received a complex lymphocyte panel and a CD4 counts	Relatively small number of cases, therefore not suitable for large scale measurement.
Screening	Colonoscopy	Proportion of persons who received a colonoscopy within 10-year interval	Building a 10-year cohort is not practical in Swiss health insurance claims database
Imaging	Radiography	1) Proportion of persons with repeated radiographies with same indication/ localisation 2) Proportion of persons with preoperative chest radiography in absence of a clinical suspicion for intrathoracic pathology	It is not feasible to evaluate the appropriateness of radiography in the claims data. Not specific to primary care. Limited influence of the GP to influence the radiography process of other health care providers.
Imaging	Ostodensitometry	Proportion of persons received repeated osteodensitometry	Relatively small number of cases, therefore not suitable for large scale measurement.. Clinical information missing.
Geriatric care	NSAIDs (≥65 years and older)	Proportion of persons with NSAID prescriptions	Complex clinical situations/ multimorbidity limit alternative therapies. Individual case review is needed.
Drug safety	Potentially inappropriate use of antibiotics	1) Proportion of persons with ≥1 antibiotic prescription 2) Proportion of women with ≥1 chinolone prescription 3) Proportion of women with ≥1 chinolone prescription who had no urine test	Interpretation without clinical information impossible. Very helpful for decision makring in clinical practice, but not appropriate for aggregated measurement.
Drug safety	Drug interaction	Proportion of persons with selected adverse drug interactions based on DEGAM S1 list	Heterogeneity in definitions/ lack of broadly accepted list of medication combinations. Low practical relevance and relatively small number of cases, therefore not suitable for large scale measurement.

*NSAID* Non-steroidal anti-inflammatory drug, *GP* General practitioner, *PIM* Potentially inappropriate medications

The results of the feasibility test were discussed in a second workshop. According to expert consensus 10 candidate indicators failed the feasibility test and were thus excluded:

Two candidates revealed too small case numbers and were thus not suitable for large-scale measurement (“DEXA-Scan” and “Complex lymphocyte panel”). In addition, “drug interaction” lacked a broadly accepted and clearly defined list for precise definition of inappropriate medication combinations. For “colonoscopy”, the recommended screening interval is 10 years. This candidate indicator was excluded because analysis of a 10-year cohort is not practical in Swiss health insurance claims dataset as Swiss residents have the possibility to change their health insurance annually. “Radiography” and “Medication after hospital discharge” were excluded because the indicators did not primarily target quality of primary care and the influence of the primary care physician is generally limited. Other candidate indicators did not pass expert discussions because there is no general negative recommendation against the underlying medical services in the general population and appropriateness of indication depends on the individual clinical situation. Therefore, according to experts, it is not appropriate to judge aspects such as “Vitamin B12 testing”, “Ferritin” or “NSAID (≥65 years and older)” based on claims data only (Table 1).

Based on discussion of current care needs in Swiss primary care, applicability and influenceability, the expert committee decided to specify the following preliminary indicators as follows: “electrolyte panel” was modified to “potassium check in patients with diuretic therapy”. To increase specificity two indicators were adapted: the indicator “arthroscopic knee intervention” was focused on patients without prior physiotherapy, and the indicator “iron infusion” was focused on persons with ≥1 iron infusion and without prior oral iron therapy.

For two of the consented new indicators relating to drug safety, the expert committee recommended to develop a pragmatic approximation of the methodology developed in previous studies using Swiss health insurance claims as a basis for further operationalisation before implementation in practice: “potentially inappropriate opioid prescription” [18] and “potentially inappropriate proton pump inhibitor prescription” [19].

In conclusion, based on informal consensus, the experts passed a final set of 9 additional new QI including of 9 additional QI covering the domains general integrated care (2 QI), efficiency (1 QI), laboratory testing (2 QI), osteoarthritis (1), and drug safety (3) (Table 2).

**Table 2:** Final set of additional quality indicators resulting from consensus process

Nr.	Category	Subject	Nominator	Denominator	Comments
1	General aspects/ efficiency	Contiuity of care (UPC index)	Sum of insured persons with consultation at the regular GP	Sum of insured persons with consultation (total: regular GP, GP, specialist)	Continuity of care threshold: Low (< 0.75), High (≥0.75); Only insured persons with ≥3 consultations were considered due to potential bias with small number of consultations. Additional option: Sum of insured persons with consultation at the regular GP/ Sum of insured persons with consultation at the GP
2	General aspects/ efficiency	Management continuity between hospital and GP (among persons ≥65 years)	Sum of insured persons aged 65 year or older who encountered a GP within 4 weeks after hospital discharge	Sum of insured persons aged 65 year or older who were discharged from hospital	Supplementary material: Sum of insured persons aged 65 year or older who encountered a healthcare provider within 4 weeks after hospital discharge/ Sum of insured persons aged 65 year or older who were discharged from hospital
3	General aspects/ efficiency	Prescription ratio of biosimilars	Sum of insured persons with biosimilar prescriptions	Sum of insured persons with biosimilar or biological prescriptions	
4	Laboratory testing	Vitamin D testing	Sum of insured persons who received Vitamin D testing	Sum of insured persons	Additional option: Sum of insured persons who received multiple/ repeated Vitamin D test / Sum of insured persons ≥1 Vitamin D test
5	Laboratory testing	Potassium check during diuretic therapy	Sum of insured persons aged 75 with loop diuretic/thiazide prescriptions who received a potassium check within a year	Sum of insured persons aged 75 with loop diuretic prescriptions	
6	Osteoarthritis	Arthroscopic knee intervention without prior physiotherapy	Sum of insured persons without physiotherapy 6 months prior to arthroscopic knee intervention	Sum of insured persons with knee arthroscopy	Additional option: Sum of insured persons who had GP consultation of knee imaging 6 months prior to knee arthroscopy/ Sum of insured persons with knee arthroscopy Cave: Exclude accidents from the analytic study sample.
7	Drug safety	Potentially inappropriate use of proton pump inhibitor	Sum of insured persons with Potentially inappropriate use of proton pump inhibitor prescription	Sum of insured persons	Operationalization is based on Muheim et al. (2021)
8	Drug safety	Potentially inappropriate use of opioids	Sum of insured persons with potentially inappropriate opioid prescription	Sum of insured persons	Operationalization is based on Wertli et al. (2017)
9	Drug safety	Iron infusion without prior diagnostics and oral treatment	Sum of insured persons who received an iron infusion without receiving 1 month prior a ferritin test and an oral iron therapy	Sum of insured persons who received an iron infusion	Relevance in women much higher than in men. Additional option: Sum of insured persons who received an iron infusion without prior ferritin test and/or oral iron therapy

*UPC* Usual Provider Continuity Index

As for the two pre-existing diabetes indicators relating to control of lipid and kidney values stakeholders raised concern that the original definition of the indicator to be calculated in all persons with antidiabetic medication irrespective of current comedication might lead to disincentives. Systematic review of guidelines for the management of diabetes revealed that none of the guidelines contained explicit recommendations on testing depending on comorbidities, comedication, or patient subgroups. The expert groups intensively discussed the topic at both workshops taking controversial evidence of statin therapy for prevention of cardiovascular events in elderly patients and current outcome measurement principles in disease management programs into account. Discussion resulted in consensus that both indicators should be adapted as follows: indicator #19 should be limited to those below the age of 76 and those without current statin therapy. Indicator #20 should be restricted to those without current therapy with angiotensin converting enzyme inhibitors or angiotensin 2 receptor antagonists (Table 3).

**Table 3:** Update of pre-existing diabetes quality indicators resulting from consensus process

Number of QI	Category	Subject	Nominator	Denominator
19	Diabetes mellitus	Proportion of insured persons **below the age of 76** with antidiabetic medication **without statin medication** receiving control of lipid values per year	Sum of insured persons with the Pharmacy Cost Group “diabetes mellitus” **below the age of 76 without statin medication** for which control of lipid values was reimbursed per year	Sum of insured persons with the Pharmacy Cost Group “diabetes mellitus” **below the age of 76 without statin medication** per year
20	Diabetes mellitus	Proportion of insured persons with antidiabetic medication **without ACE or AT2 inhibitors** receiving control of kidney values per year	Sum of insured persons with the Pharmacy Cost Group “diabetes mellitus” **without ACE or AT2 inhibitors** for which control of kidney values was reimbursed per year	Sum of insured persons with the Pharmacy Cost Group “diabetes mellitus” **without ACE or AT2 inhibitors** per year

In bold: text added to the original definition. *ACE* angentensin converting enzyme; AT2: angiotensin 2 receptor

## Discussion

This study presents 9 additional evidence-based measures for quality of primary ambulatory care in Switzerland applicable on pre-existing and nationwide available data. These new indicators extend the previously developed initial set of 24 set which has been principally well received and helped to launch discussion between stakeholders about how to increase quality of ambulatory care. Update of two established diabetes process indicators is likely to increase their relevance for subsequent care.

Quality circles are needed not only for care processes but also for methods of quality assessment [20, 21]. Currently four of these 24 QI relate to diabetes mellitus and have been implemented in pay-for-performance (P4P) contracts between networks of primary care physicians and a Swiss health insurance [7]. The specification of two of these indicators is likely to increase relevance of the underlying diagnostic processes for the subsequent care management of diabetes patients and reduce disincentives. Moreover, it may increase acceptance of such measures when both the underlying evidence base and experiences and concerns of those involved in everyday care of these patient groups are regularly reviewed and included in a continuous quality process of methodological instruments [22].

The present project is an illustrative example of a collaborative approach between practitioners, researchers, experts for local care needs and methodologists bringing together different experiences, perspectives and skills. The QI are developed based on an established and pragmatic consensus process based on international evidence and local public health needs [23, 24]. It demonstrates that bottom-up initiatives have the potential to result in practical, implementable, and continuously enhanced tools for quality improvement.

Principally, the present 24 pre-existing and 9 additional indicators complement other initiatives and data sources to monitor quality of ambulatory care such as the «Family medicine ICPC Research using Electronic medical records» (FIRE) initiative [4], the Swiss Primary Care Active Monitoring (SPAM) instrument, 56 indicators related to the organization of primary care in Switzerland [25], or quality indicators that are currently under development for the longterm nursing care setting [8].

Several limitations need to be considered. Firstly, indicators were discussed and chosen from the perspective of Swiss mandatory basic health insurance. Therefore, care measures usually performed outside of the basic health insurance package were not systematically addressed in this project (e.g. services covered by supplementary insurance, over the counter medication, or health promotion). However, the Swiss mandatory basic health insurance covers a very broad range of services needed for management of illness, accidents, and motherhood deemed to be effective, appropriate and cost-efficient [26]. Secondly, we had to systematically exclude all aspects of quality that were not included in the billing system of basic health insurance in Switzerland. Therefore, quality dimensions such as patient satisfaction, quality of life, symptoms, indications or clinical outcomes need to be addressed elsewhere. This also applies to data routinely collected in clinical settings but not transmitted to health insurances such as ambulatory hospital data. Thirdly, the underlying evidence base might systematically under- or overrepresent certain care aspects depending on the presence or absence of evidence. Finally, data for feasibility testing came from a single health insurance, and results might differ when including data from other health insurances. However, the Helsana Group covers about 15% of the Swiss population from all patient groups and Swiss regions. Previous studies showed that the population is largely representative for the general population of Switzerland, and that feasibility testing based on this data is appropriate [27–29].

The present study has implications for research. Firstly, future studies are needed to assess the level of quality in Switzerland based on the presented additional QI. Secondly, the effects of updating two of the diabetes QI on behaviour of physicians, patient outcomes and costs in the context of care regimented in contracts between Swiss physician networks and health insurances needs to be evaluated. Thirdly, future scientific efforts are needed to explore how QI based on health insurance claims data might be enriched with important information systematically lacking in health insurance claims such as patient relevant and patient reported outcomes [30].

## Conclusions

Additional evidence-based QI relating to overuse and intersectoral care aspects extend the options of measuring quality of primary care in Switzerland based on health insurance claims data and complement the initial QI set. An update of two established indicators relating to preventive measures in diabetes patients increases their relevance for subsequent care.

## Data Availability

The datasets analysed during the current study are not publicly available because they are part of the confidential Helsana health insurance claims database. Additional information not included in the paper is available from the corresponding author on reasonable request.

## Abbreviations

GP: General practitioner
NSAID: Nonsteroidal anti-inflammatory drug
PCG: Pharmacy Cost Group
PIM: Potentially inappropriate medication
QI: Quality indicator
